# Cytoreductive Surgery and Hyperthermic Intraperitoneal Chemotherapy in the Management of Colorectal Cancer with Peritoneal Metastasis: A Single-Center Cohort Study

**DOI:** 10.3390/medicina60071058

**Published:** 2024-06-27

**Authors:** Fabrizio D’Acapito, Massimo Framarini, Daniela Di Pietrantonio, Francesca Tauceri, Valentina Zucchini, Eleonora Pozzi, Leonardo Solaini, Giorgio Ercolani

**Affiliations:** 1 General and Oncologic Surgery, Morgagni-Pierantoni Hospital, AUSL Romagna, Via Forlanini 34, 47121 Forlì, Italydaniela.dipietrantonio@auslromagna.it (D.D.P.); francesca.tauceri@auslromagna.it (F.T.); leonardo.solaini2@unibo.it (L.S.); giorgio.ercolani2@unibo.it (G.E.); 2 Department of Medical and Surgical Sciences, University of Bologna, Via Zamboni 33, 40126 Bologna, Italy; valentina.zucchini4@studio.unibo.it (V.Z.); eleonora.pozzi3@studio.unibo.it (E.P.)

**Keywords:** hyperthermic intraperitoneal chemotherapy, peritoneal metastases, colorectal cancer, cytoreductive surgery, outcome

## Abstract

Multimodal treatment in peritoneal metastases (PM) from colorectal neoplasms may improve overall survival (OS). In this study, we reported our experience in using cytoreductive surgery (CRS) combined with intraperitoneal chemohyperthermia (HIPEC) for the treatment of peritoneal metastases (PM) from colorectal neoplasms. The first aim was to evaluate the overall survival of these patients. Furthermore, using the results of the Prodige 7 Trial and incorporating them with the entropy balance statistical tool, we generated a pseudopopulation on which to test the use of CRS alone. We performed a retrospective analysis based on a prospective database of all 55 patients treated with CRS + HIPEC between March 2004 and January 2023. The median OS was 47 months, with 1-, 3- and 5-year survival rates of 90.8%, 58.7% and 42.7%, respectively. There was no significant difference in the data in the pseudogroup generated with entropy balance. This finding confirms the critical role of complete cytoreduction in achieving the best OS for patients with PM. PCI > 6 seems to be the most important prognostic factor influencing OS. At present, CRS + HIPEC seems to be the therapeutic strategy that guarantees the best results in terms of OS for patients with relatively low PCI and in whom a CCS ≤ 1 can be achieved.

## 1. Introduction

Colorectal cancer (CRC) ranks third in terms of incidence worldwide and second in terms of mortality [1]. When diagnosed early, approximately 80% of patients are eligible for surgery with curative intent [2]. Colorectal cancer (CRC) ranks third in terms of incidence worldwide and second in terms of mortality [1] Half of these patients present with metastases at diagnosis, sometimes multifocal [3,4]. The most likely sites for CRC to metastasize are the liver and the lungs [5], and the peritoneum is also a common site for CRC metastasis. The site of metastasis impacts patient prognosis. The peritoneum, among the three mentioned, is the most prognostically unfavorable site [6].

As a result, in the eighth edition of the TNM staging system, peritoneal metastases (PM) were classified as M1c since they have a worse prognosis when compared with patients with one distant organ metastasis (M1a) and those with more than one distant organ metastasis (M1b) [7]. The occurrence of secondary lesions limited to the peritoneal level is observed in 25–35% of patients, but they are frequently associated with lymph node, liver and lung metastases [8].

The development of more effective chemotherapy drugs, the advancement of loco-regional treatment systems and improvements in surgical techniques and perioperative management have progressively extended the boundaries of treatment possibilities in these patients. Among the therapeutic strategies explored since the nineties, cytoreductive surgery (CRS) associated with intraperitoneal chemotherapy is the one that has, up to now, guaranteed the best results in selected patients [9].

CRS, codified by Sugarbaker, entails excision of the visceral and parietal peritoneum with the goal of clearing all visible disease. The involvement of the visceral peritoneum frequently requires the resection of portions of the stomach, small intestine, or colorectum. In the same way, small tumor nodules can be excised from the peritoneum by “electroevaporation” [10]. HIPEC can be performed using different approaches, with the two main ones being open abdomen, as originally proposed by Sugarbaker, and closed abdomen. In open abdomen, the abdominal wall is lifted according to the “Coliseum technique”, while in closed abdomen, there is temporary skin closure after inserting the inflow and outflow catheters [11].

Notably, in 2016, CRS combined with Hyperthermic Intraperitoneal Chemotherapy (HIPEC) for the treatment of oligometastatic disease was added to the ESMO guidelines [12]. To identify patients eligible for this treatment strategy, evaluation of all cases by a multidisciplinary oncology team is mandatory [13].

The recently published results of the Prodige 7 trial confirmed the essential role of CRS but did not demonstrate an additional benefit driven by the use of HIPEC according to the Elias schedule [14]. The findings of this study have enlivened the debate in the scientific community on this topic [15,16].

The goal of our study is to evaluate the outcomes of CRS + HIPEC procedures for PM from colorectal malignancies performed in an Italian medium-volume center with a dedicated multidisciplinary oncology team.

## 2. Materials and Methods

We conducted a retrospective review of a prospectively collected database including all patients who underwent CRS/HIPEC for CRC-PM at the Morgagni-Pierantoni Hospital in Forlì between March 2004 and January 2023. The diagnostic–therapeutic protocol and data collection were approved by CEROM, Hospital Ethics Committee Protocol code 0/23453/F2RP (date of approval 4 June 2004), and written informed consent was signed by all patients. CRS plus HIPEC was offered to patients younger than 76 years of age with an ASA1-3, and a procedure was performed for both synchronous and metachronous PM from colorectal neoplasm. PM from appendicular neoplasms was excluded from this study. Non-resectable extra-peritoneal metastatic disease and nodal disease outside the primary field was a contraindication to treatment. As a derogation from this rule, patients with limited and stable metastatic lung disease since 2019 were also enrolled in the protocol. The presence of liver metastases fewer than 4 in number and resectable by simple resections has never been considered a contraindication to CRS and HIPEC. At our center, the possibility of re-HIPEC is available.

All patients were discussed at a specialized peritoneal tumor multi-disciplinary team (MDT) meeting with CRS/HIPEC offered as a treatment with curative intent. The MDT consisted of at least one oncologist, one dedicated radiologist, two dedicated surgeons, an endoscopist and, in selected cases, a thoracic surgeon or an HPB or urologist. The preoperative study included a CT scan of the chest–abdomen with contrast medium for all patients. In cases of lesions with no definite interpretation in the liver, lungs or pelvis, the patient underwent an MRI or PET scan. If bladder or ureteral involvement was suspected, a urologic assessment with cystoscopy was performed and, if necessary, ureteral stent placement was scheduled at the beginning of the surgical procedure.

The chemotherapy protocol included oxaliplatin as the first choice (in accordance with the Elias protocol [17,18]) during HIPEC and a scheme with Mitomycin C as an alternative. The choice of HIPEC agent was decided by specialist medical oncologists who were core members of the peritoneal tumor MDT. In all cases, this decision involved a review of the patients’ health record and treatment history, and toxicities to previous systemic anticancer treatments.

### 2.1. Surgical Technique

The patient was placed in the lithotomy position. Access to the peritoneal cavity was through a xipho-pubic laparotomy.

Peritoneal disease burden was calculated using the peritoneal cancer index (PCI) score, a semi-quantitative indicator of the extent of PM lesions, proposed by Sugarbaker [19,20]. CRS included removal of the primitive neoplasm; if still in place, removal of the omentum; and the resection of all peritoneal implants by combining visceral resection (when necessary) and peritonectomy. The completeness of cytoreduction was evaluated in accordance with the complete cytoreduction score (CCS) [20]. Until 2014, HIPEC was performed before performing any bowel anastomoses; subsequently, anastomoses were performed earlier.

HIPEC was administered using a semiclosed colosseum technique [21] according to the Elias protocol: intravenous 5-fluorouracil (400 mg/m^2^) with folic acid (20 mg/m^2^) and intraperitoneal oxaliplatin (460 mg/m^2^) for 30 min at 41.5 °C or Mitomicina C (3.4 mg/m^2^). During the hyperthermia phase, each patient’s diuresis was stimulated to ensure the production of at least 10 cc/minute of urine, and amifustine was routinely administered as a renal function protector until 2020. According to protocol, a transfusion of fresh frozen plasma was provided at the end of the HIPEC phase. All patients at the end of surgery were moved to the intensive care unit for the first 24–48 h.

All patients had their pathology reports, operation notes and hospital records reviewed. Patient demographics and treatment history (prior surgery or chemotherapy) were extracted. The operative data included the date of the CRS/HIPEC procedure, PCI and CCS at CRS/HIPEC. The pathological assessment included the features of the primary tumor, mucinous components and/or signet ring cell morphology, the degree of differentiation and the presence of nodal metastases (N-stage).

Patients were followed up every 6 months for 2 years after CRS/HIPEC and annually thereafter, with computed tomography (CT) of the thorax/abdomen/pelvis at 6, 12, 18, 24, 36, 48 and 60 months, accompanied by tumor markers.

### 2.2. Statistical Analysis

The primary outcome measure was overall survival (OS) from the time of CRS/HIPEC.

Common descriptive analyses were carried out using chi-square, Fisher’s exact and Mann–Whitney U tests.

A Kaplan–Meier curve was used to calculate survival rates, and differences in survival between subgroups were assessed by means of a log rank test. Overall survival (OS) was defined as the time between diagnosis and death/the last follow up. Disease-free survival (DFS) was defined as the time between surgery and the first evidence of disease recurrence.

To compare our results with a control group (no HIPEC), a pseudopopulation weighted on the control group of the Prodige7 trial [22] was created using “entropy balance”. The latter is a data preprocessing procedure that allows reweighting of a dataset so that the covariate distributions in the reweighted data satisfy specified conditions [17]. In other words, reweighting allowed us to estimate what would have happened if the patients treated with HIPEC had received the same treatment without it. Analyses were performed with STATA22, using the ebalance command.

## 3. Results

**Figure 1:**
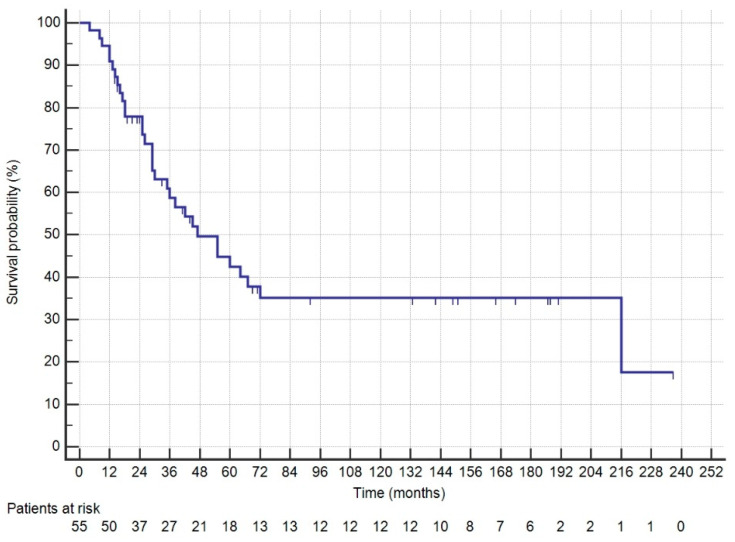
OS of all patients who underwent CRS + HIPEC.

**Figure 2:**
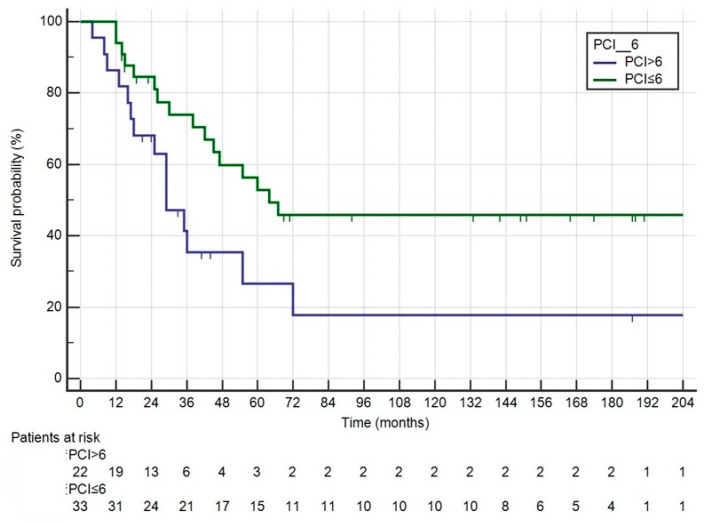
OS of all patients undergoing CRS + HIPEC categorized into two groups according to PCI > 6 and PCI ≤ 6.

**Figure 3:**
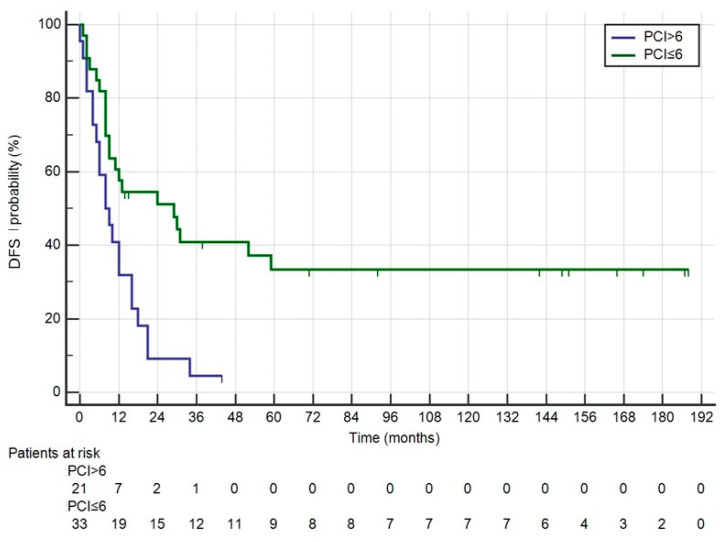
DFS of all patients undergoing CRS + HIPEC divided into two groups according to PCI > 6 and PCI ≤ 6.

**Figure 4:**
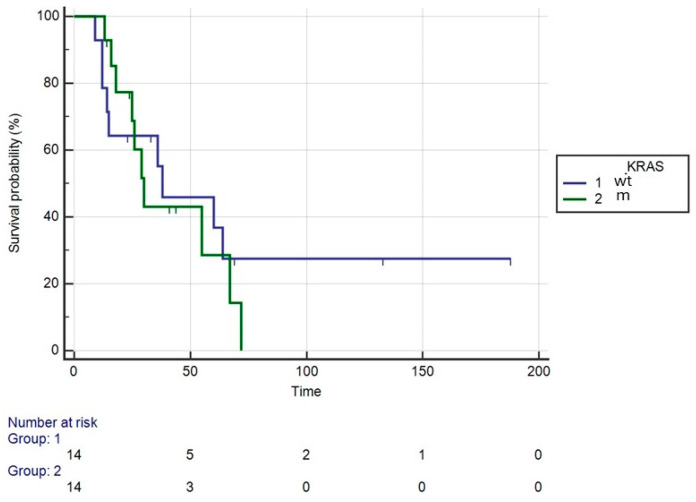
OS of all patients undergoing CRS + HIPEC categorized into two groups according to KRAS wild type (WT) or mutated (M).

The patients’ characteristics are shown in Table 1. Fifty-five patients underwent CRS + HIPEC, with Mitomycin C used as the intraperitoneal drug in the first HIPEC for two cases. Three patients underwent a second CRS + HIPEC procedure, again using Mitomycin C. Median survival was 47 months (range: 30–72), with a median follow-up of 147 months (range: 92–187). The 1-, 3- and 5-year survival rates were 90.9%, 58.7% and 42.7%, respectively (Figure 1). The median DFS was 12 months (range: 8–21). Recurrence involved the peritoneum in 37 patients (67.27%), with 32 cases involving multiple sites, as detailed in Table 2. Grade > 2 complications occurred in nine patients (16.36%), and reoperation was necessary in three cases: two for evisceration due to dehiscence of the midline laparotomy, and one for an ileal perforation recognized 7 days post-CRS. The median length of postoperative hospital stay (LOS) was 15 days. For all patients, the median interval between the diagnosis of PC and surgery was 120 days. For metachronous cases, the interval between the diagnosis of carcinomatosis and surgical treatment was 180 days. For patients receiving adjuvant chemotherapy, surgery was performed within 60 days; the interval increased to 210 days for patients who underwent neoadjuvant chemotherapy for carcinomatosis. PCI > 6 was the only factor affecting OS and DFS (Figure 2 and Figure 3). KRAS status was evaluated in 28 patients, with 14 being wild-type (WT) and 14 mutated (Mt) (Figure 4). BRAF status was evaluated in 18 patients, with 13 WT and 5 Mt. Entropy balance assessment was performed in 52 patients who met the criteria for the Prodige7 study. Patients with PCI ≥ 25 and those who had the first HIPEC with Mitomycin C were excluded. Comparisons between the study group and the Prodige7-weighted pseudopopulation are shown in Table 1. A higher number of perioperative (pre- + post-surgery) chemotherapy treatments were observed in the control group (34, 65.4% versus 18, 34.6%; *p* = 0.008). The 1-, 3- and 5-year survival rates were 92.3%, 58.7% and 42.2% for the study group, compared to 89.1%, 56.6% and 42.5% for the pseudopopulation (*p* = 0.527).

## 4. Discussion

The OS reported in our study cohort of 47 months appears to be at least in line with major trials in the literature. Esquivel’s 2014 study of 705 patients reports an OS of 41 months [23]. Prada-Villaverde in 2014 shows, in 539 patients, an OS of 32.6 months [24]. Cashin’s [25] propensity score matching published in Lancet in 2023 (1613 patients enrolled) has an OS of 45.7 months as the best outcome (in patients who had adjuvant chemotherapy after CRS + HIPEC). Hentzen, in a 2019 study of 433 patients, reports an OS for synchronous and metachronous PM of 34 and 33 months, respectively [26]. Quénet F. in a 2021 study of the Prodige 7 trial, reports an OS of 41 months [14]. The latest multicenter study published by Fisher O.M. in 2024 [27], evaluating the largest multicenter case series collected, establishes the superiority of the Elias protocol with oxaliplatin over HIPEC protocols with Mitomycin C (OS 47 vs. 39 months). It also brings surgeons’ attention to the results of dual intraperitoneal chemotherapy (oxaliplatin + irinotecan), which raises survival to 61 months without a significant increase in morbidity, unlike previous reports in the literature.

In all the papers cited so far, HIPEC is performed with different pharmacological protocols (oxaliplatin, Mitomycin C…) and surgical modalities (open, closed, semi-closed…), even within the same study. The relative advantages of open and closed HIPEC techniques have been debated in the literature, but there still remains a lack of agreement on the best approach [28]. The first point that could be made by observing the variability in the results presented in these studies is that if CRS alone was the driver of the results, OSs should be more homogeneous with each other, regardless of the HIPEC protocol used.

We have 12 months of disease-free survival in our study cohort. When there is recurrence, it rarely involves the peritoneal site alone; however, we do not have enough data to argue about specific risk factors. Recurrence more often involves multiple sites, and in these cases, the peritoneum is almost always involved. Looking at the peritoneum as an organ (in the same way as the liver or lungs) peritoneal DFS could actually be framed as the real goal of HIPEC. The Arijona-Sanchez study published in in 2023 may be helpful in clarifying this concept, in fact this paper documents a decrease in the risk of peritoneal recurrence by up to 80% over 3 years in patients (with advanced colic neoplasia) undergoing prophylactic HIPEC [29].

The pseudopopulation generated with the entropy balance tool confirms the results of Prodige 7. If our population had not been treated with HIPEC (but only with CRS) it would have obtained the same OS results as that trial (without statistical differences).

The results of systemic chemotherapy alone in patients with PM are currently still unsatisfactory, as documented by Franko’s 2016 study [6]. Several authors have tried to define the best timing for the use of systemic chemotherapy in association with HIPEC. Tonello’s 2024 study documented an advantage for the adjuvant setting of systemic chemotherapy only relative to DFS [30].

The study by Cashin et al., 2023, suggests that there is no OS benefit to using systemic chemotherapy in a neoadjuvant setting, whereas its use in adjuvant chemotherapy results in a benefit in terms of both OS and DFS [25].

Patient selection remains the key issue. The prognostic value of PCI is clear, as is the need to achieve complete cytoreduction. In our population, where all patients had a CCS equal to 0 or 1, PCI > 6 was the only factor affecting OS and DFS.

Attempts have been made in the literature to define a PCI cut-off beyond which the procedure might not benefit the patient in terms of OS; however, agreement has not yet been achieved. In 2010, Elias proposed PCI > 20 as a relative contraindication [31]. Within the Prodige7 study, only patients with PCI > 25 were excluded from treatment [22]. Diagnosing PM early to treat patients with the lowest disease burden is the most desirable situation. The biology of the tumor and its aggressiveness play an equally important role in terms of prognosis.

Although in our case series the KRAS mutation does not appear to have an effect on OS, there are studies that have documented its impact. According to Tonello et al., 2022, patients with KRAS and BRAF mutations experiences shorter OS than wild-type patients [32], but patients with some mutations (e.g., KRAS^MUT1^) could have a similar prognosis to wild-type patients [30].

Over the past 20 years, the safety of CRS + HIPEC in terms of morbidity and mortality has progressively increased. In accordance with a systematic review by Wajekar, morbidity rates were between 12% and 60% and mortality rates between 0.9% and 5.8% [33]. The mortality and morbidity data from this study confirm what has been expressed in the recent literature. Macfie R.C. confirmed how the risk of major complications and Failure to Rescue correlated with CRS extension rather than HIPEC; in fact, in his study, there was no significant difference in Failure To Rescue between the CRS and CRS/HIPEC groups (4.0% vs. 2.3%, *p* = 0.258), nor in the pooled incidence of major complications (37.9% vs. 36.1%, *p* = 0.48) [34]. A comparative analysis showed that CRS plus HIPEC is safe, often safer across the full spectrum of NSQIP safety parameters than oncology procedures of similar risk [35].

## 5. Conclusions

At present, CRS + HIPEC seems to be the therapeutic strategy that guarantees the best results in terms of OS and DFS for PM patients from CRC with relatively low PCI and in whom a CCS ≤ 1 can be achieved. A more accurate assessment of the mutational status of the neoplasm can help surgeons select patients. The use of systemic chemotherapy in adjuvant timing seems to bring benefits compared with neoadjuvant chemotherapy. The morbidity related to CRS + HIPEC is currently comparable to that of other complex cancer surgery procedures.

## Figures and Tables

**Table 1:** Patients’ characteristics.

	CRS + HIPEC Given to 55 Patients	CRS + HIPEC According to Prodige7 Criteria 52 Patients	Prodige7-Weighted Pseudopopulation CRS without HIPEC (*n* = 52)	*p* *
**Age (median)**	61	61.2	59 (50–66)	0.510
**Sex**				0.844
Male	31	28	26	
Female	24	24	26	
**Primary tutor localization**				0.959
Right	20	19	20	
Transverse	2	2	3	
Left	29	27	25	
Rectum	4	4	4	
No specific info	0	0	0	
**Synchronous peritoneal metastases**	13	12	12	
**Previous surgery**				0.856
For primary tumor	35	33	40	
For peritoneal metastases	7	7	8	
**Previous cht**				0.662
For primary tumor	28	27	25	
For peritoneal metastases	0	0	0	
OX in cht schedule	22	19	23	
**Systemic cht-HIPEC**				0.008
No cht	6	6	2	
Pre-operative	11	10	9	
Post-operative	18	18	7	
Both	20	18	34	
**CCS**				1.000
CC0	53	52	52	
CC1	2	0	0	
**PCI**				0.070
Median	6 (1–26)	5.5 (1–21)		
<11	40	40	30	
11–15	10	10	21	
>15	5	2	2	
**Time from diagnosis of PM to surgery, days (median)**	120	120	210 (60–330)	0.042
**Duration of surgery (min, median)**	620	620	620 (540–680)	0.865
**LOS (days, median)**	15	15	14 (12–18)	
**Re-intervention**	3	3	3	1.000
**Mortality**	0	0	0	1.000
**Clavien–Dindo > 2**	9	9	7	0.786

* Comparison between Prodige7 criteria group and pseudopopulation. CCS: complete cytoreduction score; cht = chemotherapy; LOS = length of hospital stay; OX = oxaliplatin; PCI = peritoneal cancer index; PM = peritoneal metastases.

**Table 2:** Recurrence sites.

Sites of Recurrence	Pts	%
No recurrence	13	23.8
Peritoneum only	4	7.2
Peritoneum + other sites	33	60
Liver only	1	1.8
Lung only	1	1.8
Nodes only	1	1.8
Multiple sites without peritoneum	2	3.6

## Data Availability

The data presented in this study are available upon request to the corresponding author due to limitations related to the protection of privacy.
